# Supplementary material for: Resistance and Co-Resistance of Metallo-Beta-Lactamase Genes in Diarrheal and Urinary-Tract Pathogens in Bangladesh
Source: Microorganisms. 2024 Aug 5;12(8):1589. doi: 10.3390/microorganisms12081589 (PMC11356267; doi:10.3390/microorganisms12081589)
Supplement: Supplementary file 1 [file microorganisms-12-01589-s001.zip › Figure S1-Etiology.pdf]

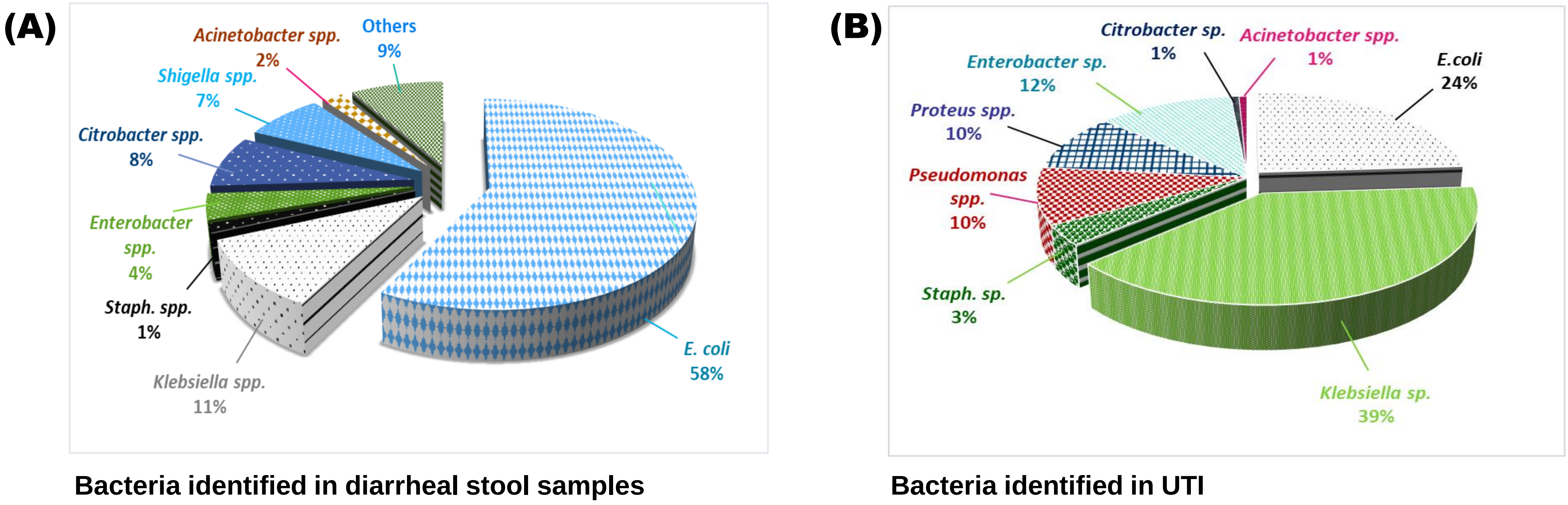

Supplementary Figure S1. A) Different bacteria isolated and identified from diarrheal stool specimens. B) Bacteria isolated and identified from urinary tract infections. .
