# Supplementary material for: Resistance and Co-Resistance of Metallo-Beta-Lactamase Genes in Diarrheal and Urinary-Tract Pathogens in Bangladesh
Source: Microorganisms. 2024 Aug 5;12(8):1589. doi: 10.3390/microorganisms12081589 (PMC11356267; doi:10.3390/microorganisms12081589)
Supplement: Supplementary file 1 [file microorganisms-12-01589-s001.zip › Figure S2-Comparative Suceptibility.pdf]

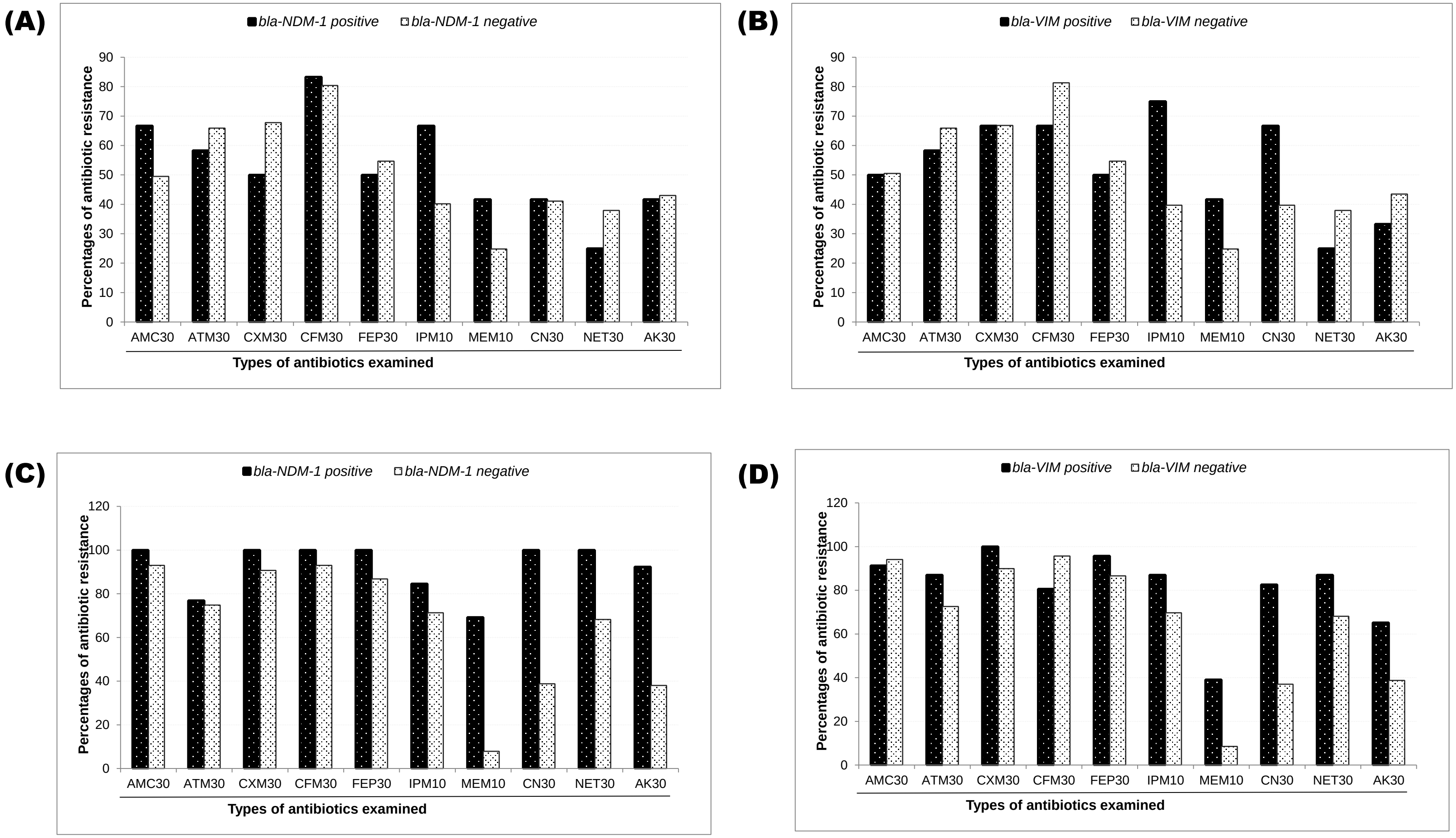

Supplementary Figure S2. Impact of two metallo-β-lactamase genes (MBL) to the phenotypic susceptibilities of β-lactam, carbapenem, and aminoglycoside antibiotics. The susceptibilities of the MBL-positive and -negative isolates were assessed against ten antibiotics, namely amoxycillin-clavulanic acid (AMC 30 μg), aztreonam (ATM 30 μg), cefuroxime sodium

(CXM 30 μg), cefixime (CFM 30 μg), cefepime (FEP 30 μg), imipenem (IMP 10 μg), meropenem (MEM 10 μg), gentamicin (CN 30 μg), netilmicin (NET 30 μg), and amikacin (AK 30 μg). (A). The Y-axis values of black-spotted bars indicate the percentage of *bla*NDM-1 gene-carrying diarrheal pathogen showing as resistance against respective antibiotics shown on the X-axis. Similarly, the white bars illustrate the percentages of resistance isolates that do not carry the *bla*NDM-1 gene. Resistance of (B) *bla*VIM-positive (n=12) and -negative (n=226) diarrheal pathogens, (C) *bla*NDM-1-positive (n=13) and -negative (n=129) UTI pathogens, and (D) *bla*VIM-positive (n=23) and -negative (n=119) UTI isolates are presented separately.
