# Supplementary material for: Resistance and Co-Resistance of Metallo-Beta-Lactamase Genes in Diarrheal and Urinary-Tract Pathogens in Bangladesh
Source: Microorganisms. 2024 Aug 5;12(8):1589. doi: 10.3390/microorganisms12081589 (PMC11356267; doi:10.3390/microorganisms12081589)
Supplement: Supplementary file 1 [file microorganisms-12-01589-s001.zip › Supplementary Figure S3. MAR Index.pdf]

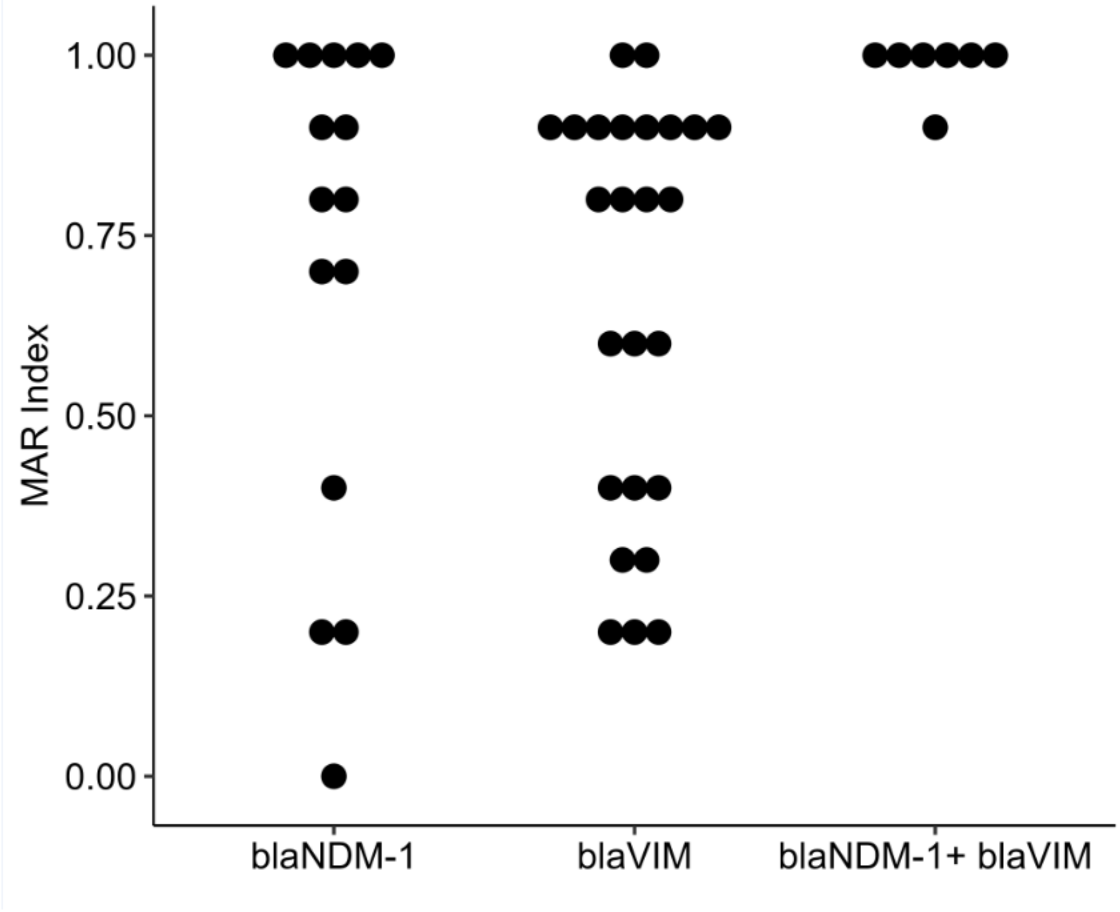

Supplementary Figure S3. Multiple antibiotic resistance (MAR) index of single carrying and co-carrying metallo-β-lactamase (MBL) genes. The multiple antibiotic resistance (MAR) index was calculated by dividing the number of antibiotics an isolate was resistant by the total number of antibiotics tested. The dot plot shows the MAR index values of the pathogens that carried a single MBL gene exclusively (either *bla*NDM-1 or *bla*VIM) and concurrently (both *bla*NDM-1 and *bla*VIM together) in the diarrheal pathogens and uropathogens. The MAR index values were placed on the Y-axis, and the group of MBL-gene carriage was placed on the X-axis. Each dot represents the MAR index value of one bacterial pathogen.
