# Supplementary material for: Resistance and Co-Resistance of Metallo-Beta-Lactamase Genes in Diarrheal and Urinary-Tract Pathogens in Bangladesh
Source: Microorganisms. 2024 Aug 5;12(8):1589. doi: 10.3390/microorganisms12081589 (PMC11356267; doi:10.3390/microorganisms12081589)
Supplement: Supplementary file 1 [file microorganisms-12-01589-s001.zip › Supplementary Table S1.pdf]

Supplementary Table S1. Phenotypic-genotypic association of mettalo β-lactamase (MBL) genes and β-lactam antibiotic resistance.

Phenotypic susceptibility		Presence of MBL genes in diarrheal and UTI isolates (n=370)					
		bla-VIM, no (%)		P value	*bla-NDM-1, no (%)*		P value
		Positive (n=35)	Negative (n=335)		Positive (n=25)	Negative (n=345)	
AMC 30	Sensitive	8 (22.9)	115 (34.3)	.191	4 (16.0)	119 (33.5)	.077
	Resistant	27 (77.1)	220 (65.7)		21 (84.0)	226 (65.5)	
ATM 30	Sensitive	8 (22.9)	107 (32.1)	.338	8 (32.0)	107 (31.2)	1.0
	Resistant	27 (77.1)	226 (67.9)		17 (68.0)	236 (68.8)	
CXM 30	Sensitive	4 (11.4)	85 (25.4)	.094	6 (24.0)	83 (24.1)	1.0
	Resistant	31 (77.0)	250 (74.6)		19 (76.0)	262 (75.9)	
CFM 30	Sensitive	5 (14.3)	50 (14.9)	1.0	2 (8.0)	53 (15.4)	.558
	Resistant	30 (85.7)	285 (85.1)		23 (92.0)	292 (84.6)	
FEP 30	Sensitive	7 (20.0)	115 (34.3)	.097	6 (24.0)	116 (33.6)	.384
	Resistant	28 (80.0)	220 (65.7)		19 (76.0)	229 (66.4)	
IMP 10	Sensitive	6 (17.1)	167 (49.9)	**.000**	6 (24.0)	167 (48.4)	**.022**
	Resistant	29 (82.9)	168 (50.1)		19 (76.0)	178 (51.6)	
MEP 10	Sensitive	21 (60.0)	270 (81.1)	**.007**	11 (44.0)	280 (81.6)	**.000**
	Resistant	14 (40.0)	63 (18.9)		14 (56.0)	63 (18.4)	

%, column percentage; p values of statistically significant associations are shown bold.

AMC 30, amoxycillin-clavulanic acid 30 μg; ATM 30, aztreonam 30 μg; CXM 30, cefuroxime sodium 30 μg; CFM 30, cefixime 30 μg; FEP 30, cefepime 30 μg; IMP 10, imipenem 10 μg; MEM 10, meropenem 10 μg.
